# Perceived mental health in parents of children with rare congenital surgical diseases: a double ABCX model considering gender

**DOI:** 10.1186/s13023-021-01998-9

**Published:** 2021-09-09

**Authors:** Johannes Boettcher, Holger Zapf, Mareike Fuerboeter, Rojin Nazarian, Konrad Reinshagen, Silke Wiegand-Grefe, Michael Boettcher

**Affiliations:** 1 Department of Child and Adolescent Psychiatry, Psychosomatics and Psychotherapy, University Medical Center Hamburg-Eppendorf, Martinistrasse 52, 20246 Hamburg, Germany; 2 Department of Pediatric Surgery, University Medical Center Hamburg-Eppendorf, Martinistrasse 52, 20246 Hamburg, Germany

**Keywords:** Mental health, Rare diseases, Parents, Pediatric surgery, Double ABCX model

## Abstract

**Background:**

Previous research has supported the utility of the Double ABCX model of family adaptation for parents in various diseases. Nonetheless, it remains unclear how raising a child with rare congenital surgical diseases impacts the mental health of both mothers and fathers.

**Methods:**

The potential predictors of maternal and paternal mental health in a German sample of 210 parents of children with rare congenital surgical diseases were investigated. Parents were investigated cross-sectionally utilizing standardized psychometric questionnaires that assessed factors attributed to parental adaptation within the Double ABCX model.

**Results:**

Stressor pile-up, family functioning, perceived stress, and mental health were positively associated with mothers and fathers. However, further analyses revealed that family functioning, social support, and perceived stress fully mediated the positive association between stressor pile-up and mental health in mothers, but not fathers.

**Conclusion:**

Our findings suggest that parental adaptation to a rare congenital surgical disease in their children may be improved by increased intra- and extrafamilial resources and decreased perceived family-related stress in mothers, but not fathers. Our results may help to identify gender-specific factors that may guide clinicians and future interventions.

## Background

Rare diseases are defined by a prevalence of less than 1:2000 in the European Union [1]. It is estimated that more than 7000 different rare diseases affect about 13.5–25 million people in Europe [2]. Even though parents of children with rare diseases show a reduced Quality of Life [3] and increased mental health symptoms [4–6], in general, those parents have received little attention from the research and medical community [7].

A specific subgroup of rare diseases requires surgical treatment early on. These rare diseases include anorectal malformation, biliary atresia, congenital diaphragmatic hernia, esophageal atresia, and Hirschsprung’s disease. Caused by the enormous heterogeneity and sustainable variation in severity, some defects can be easily repaired, but others require multiple surgeries, which potentially go along with life-long illness and chronic disability [8]. Following the diagnosis of rare congenital surgical diseases, parents engage in a continuous adaptation process throughout their child’s development [9]. Similarly, parents of children with other rare diseases tend to show reduced mental health [10].

**Fig. 1:**
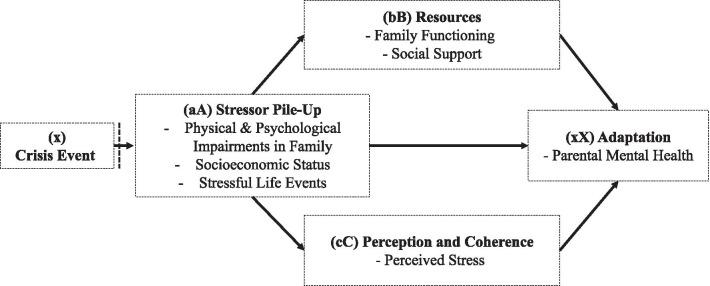
Double ABCX model. Adapted from McCubbin & Patterson (1982)

One model that can account for cumulative stressors such as different degrees of disease severity and time since diagnosis is the Double ABCX model of family stress and adaptation [11]. The respective model represents an expansion of the ABCX Model developed by Hill (1958) [12], which includes innovations in theory development and statistical modeling, opening up new pathways for research in family stress and coping [13]. The double ABCX model describes the process of adaptation to stressful events, including the following three variables are included in the double ABCX model, which is fed by the crisis event (x) [14]: The stressor pile-up (aA) represents aggregated demands resulting from the crisis event (e.g., special care needs of the child) and additional stressors occurring with time (e.g., unemployment) [11]. The intermediary factors include existing adaptive resources (bB) of the family (e.g., social support) and perception and coherence (cC) of the crisis within the family (e.g., evaluation of the stressor as a danger or a challenge). The product of the interactions between these variables yields whether a family and its members adapt (xX) positively or negatively to a crisis (Fig. 1).

In the context of rare congenital surgical diseases, a crisis event (x) is the diagnosis of a rare disease and, as given in this study, the circumstances caused by the COVID-19 pandemic. In recent research on families of children with a chronic condition, different indicators have been used to operationalize the cumulative demands forming stressor pile-up (aA), among them scales assessing stressful life events of the family [15], life stress [16], disease severity, behavioral problems of the child [17], or single items such as parental educational attainment, marital status [18], and income [19]. Adaptive resources (bB) were assessed as social or family support and family functioning or family environment [16, 18, 20]. The operationalization of perception and coherence (cC) has also varied across studies, including self-reports of sense of coherence [20], spiritual wellbeing, and perceived parental/family stress [15, 18]. Mental health, depression, and health-related quality of life [19] were used as adaptation indicators (xX).

Recent studies indicated that different outcomes in fathers and mothers of children with rare congenital surgical diseases are expected. Mothers showed worse conditions regarding mental health and quality of life, even though the father's quality of life has been found to be reduced in comparison to normative samples [21, 22].

The objective of the current cross-sectional study was to investigate factors within the Double ABCX model to explain parental mental health when raising a child with a rare congenital surgical disease. Our specific aim was to explore the fit of the ABCX model concerning the mothers and fathers in the sample. In line with previous research, we specified adaptation (xX) as mental health, resources (bB) as family functioning and social support, perception, and coherence (cC) as perceived family stress, and stressor pile-up (aA) as the cumulative demands the parents face. According to the model, we expected that the association between stressor pile-up (aA) and adaptation (xX) diminishes relevantly when resources (bB), as well as perception and coherence (cC), are integrated into the model. Concerning the parents' sex, we assumed that models would differ for mothers and fathers in accordance with previous findings.

## Methods

### Study design

Parents of children and adolescents with rare congenital surgical diseases were investigated in a quantitative-based cross-sectional study between March 2020 to April 2021 by means of standardized psychometric questionnaires. The study received ethical approval from the Medical Chamber Hamburg (PV7161) and was preregistered in ClinicalTrials.gov (NCT04382820).

### Measures

#### Stressor pile-up (aA factor)

The items for stressor pile-up were selected based on previous studies [8–10] and discussions of expected stressors with clinicians. As in previous research, dichotomous variables were summed together [18]. Also, in line with previous research and due to a lack of information on the impact of the different aspects, no weights were assigned to the single variables. The stressor pile-up count was based on the summary of the following nine dichotomized variables: (1) distance from the last surgery less than 1.5 years (n[yes] = 56/114, 49.1%); (2) presence of level of care of the affected child (n[yes] = 43/109, 39.4%); (3) presence of physical comorbidities of the affected child (n[yes] = 49/114, 43.0%); (4) parents’ non-high school education attainment in mothers (n[yes] = 32/101, 31.7%) and fathers (n[yes] = 42/96, 43.8%); (5) parents’ unemployment in the last 12 months in mothers (n[yes] = 9/107, 8.4%) and fathers (n[yes] = 2/100, 2.0%); (6) presence of a physical disease in mothers (n[yes] = 23/109, 21.1%) and fathers (n[yes] = 20/100, 20.0%); (7) presence of COVID-19 lockdown measures during the survey (n[yes] = 43/114, 37.7%); (8) parent-rated psychological distress of the affected children (SDQ total score $\ge$ 13) by mothers (n[yes] = 29/96, 30.2%) and fathers (n[yes] = 28/90, 31.1%); (9) self-reported psychological distress of the partner (BSI GSI Score $\ge$ 63) in mothers (n[yes] = 34/104, 33.3%) and fathers (n[yes] = 11/93, 11.8%).

#### Parental resources (bB factor)

The German version of the Family Assessment Measure (FAM; Cierpka & Frevert, 1994) was used to assess the family functioning of the family as a whole. It consists of 40 items, which are answered on a four-point rating scale. In addition to the seven subscales task accomplishment, role performance, communication, emotionality, affective involvement, control, and values and norms, the FAM contains the two control scales, social desirability, and defensiveness. We solely used the total FAM score of the general scale to capture the resources and problems of the families by summing up all values. Higher scores indicate worse family functioning. The FAM has shown acceptable psychometric properties [31].

The Oslo-Social Support Scale [23] is a brief checklist measuring social support. It consists of three items asking about the number of people one can rely on for personal problems, the evaluation of third parties' interest in oneself, and the possibility of getting practical support from neighbors and friends. The higher the sum score, the stronger the social support. The German version of the OSSS-3 has shown good psychometric properties [24].

#### Parental perception and coherence (cC factor)

The German version of the Impact on Family Scale (IFS) as a self-report instrument was used to measure the family-related stress due to the chronic condition and disability in childhood of the child [25]. The German scale includes 33 items on five subscales, including daily/social impact, personal impact/worries about the future, financial impact, impact on coping, and impact on siblings [26]. The items were answered on a four-point scale with higher values indicating a higher level of family-related stress. We solely used the total score that includes all items except for the dimension impact on siblings. The psychometric properties of the IFS are considered to be acceptable [26].

#### Parental adaptation (xX factor)

The Brief Symptom Inventory (BSI) was used to measure parental mental health problems [27]. The BSI includes 53 items covering somatization, compulsivity, interpersonal sensitivity, depression, anxiety, hostility, phobic fear, paranoid thinking, and psychoticism. We used the Global Severity Index (GSI) as a global index of psychological distress. The GSI is calculated using the sums for the nine symptom dimensions plus four additional items not included in any of the dimension scores and dividing by the total number of items to which the individual responded. Higher BSI scores indicate greater mental health. The German version of the BSI has been found to have good psychometric properties [28].

*Socio-demographic and clinical variables* Parents completed a study-specific socio-demographic questionnaire about their sex, age, and socioeconomic status. Clinical variables of the children included the clinical diagnoses, time since last surgery, level of care, and physical comorbidities (atopic eczema, bronchial asthma, cardiac anomalies, celiac disease, chronic cholecystitis, chronic reflux esophagitis, cleft lip and palate, clubfeet, disturbance of blood coagulation, duodenal stenosis, epilepsy, failure to thrive in obesity, renal anomalies, scoliosis, tracheobronchomalacia, urogenital anomalies, VACTERL-association). Psychosocial variables of the parents included the presence of a physical disease, the parent-rated psychological distress of affected child measured with the Strength and Difficulties Questionnaire (SDQ) total score among children aged 3–16 years, and psychological distress of the partner measures with the BSI Global Severity Index. Moreover, the presence of COVID-19 lockdown measures during the survey was included.

### Sample

Inclusion criteria for the families were as follows: (1) having a child under 21 years of age, (2) with a diagnosed rare congenital surgical disease, including anorectal malformation, biliary atresia, congenital diaphragmatic hernia, esophageal atresia, or Hirschsprung’s disease were included in the study. In addition, severe physical, mental, or cognitive impairments were set as exclusion criteria, making participation impossible or unreasonable. Signed informed consent was given by the parents. The parents were allowed to withdraw from the study at any given time.

Three hundred forty-two families with children and adolescents with rare congenital surgical diseases were identified between 2012 and 2020 in the operative registry from the Clinic of Pediatric Surgery of the University Medical Center Hamburg Eppendorf. Families were excluded due to lack of consent to participate (*n* = 3), lack of German language skills (*n* = 13), lack of contact data (*n* = 62), or not meeting the inclusion criteria (*n* = 56). Questionnaires were handed out to 208 families.

The response rate was 54.3% for families of rare diseased children. Finally, written consent from 113 families was obtained. A total number of 210 parents completed the questionnaires about parental mental health, consisting of 109 mothers and 101 fathers. 96 (84.2%) of all parent ratings were answered by both parents. All participating children met the definition of a rare disease according to the European Commission [1], and all diagnoses were verified by medical personnel.

### Statistics

Data analysis was performed using descriptive statistics (frequencies, means, and standard deviations) and bivariate tests (chi-square tests). Associations between variables were analyzed with Spearman correlations. Three variables were tested as a possible mediator of the relations between stressor pile-up and parental mental health. Multiple mediation was tested following the procedure from Preacher and Hayes (2008) [29]. The multiple mediation analyses were conducted using the PROCESS macro [30], with 10,000 bootstrapping resamples, and bias-corrected 95% confidence intervals were calculated. As an indication of effect size, *R*^2^ was used. Statistical significance was set at *p* < 0.05 (two-tailed). Statistical analyses were conducted using SPSS Statistics 26.

## Results

### Characteristics of the study population

Table 1 shows the main sociodemographic and disease characteristics. The questionnaires were completed by 210 parents. For two mothers and four fathers, items from a specific measure were missing. The final sample for analysis therefore comprised of 204 parents. Regarding the child’s age, there was a significant difference between participants (*M* = 4.2, 95% CI [3.6, 4.8], *SD* = 3.33) and non-participants (*M* = 6.5, 95% CI [6.0, 7.1], *SD* = 3.96). However, the child’s gender between participants (female = 44, male = 70) and non-participants (female = 123, male = 107) did not differ. At last, no difference between participants (anorectal malformation = 30, biliary atresia = 14, congenital diaphragmatic hernia = 14, esophageal atresia = 27, Hirschsprung’s disease = 29) and non-participants (anorectal malformation = 65, biliary atresia = 20, congenital diaphragmatic hernia = 27, esophageal atresia = 40, Hirschsprung’s disease = 76) could be found regarding the disease-groups.

**Table 1:** Sociodemographic and disease characteristics of the respondent

	Participants (*n* = 114 families)	
Characteristics	*M*	*SD*
Patient’s age (years)	4.2	3.33
Mother’s age (years)	37.4	5.98
Father’s age (years)	40.3	6.15
Number of surgeries due to disease	4.4	4.21
Time since first surgery (years)	2.6	2.51
Time since initial diagnosis (years)	3.8	3.04

Parents	*n*	%
Gender	109/101	95.6/88.6
Marital status (mothers/fathers)		
Married/living together	98/92	89.9/91.1
Single	9/7	8.3/6.9
Divorced	2/2	1.8/2.0
Not stated	0.0/0.0	0.0/0.0
Education (mothers/fathers)		
Lower-middle education	32/42	29.4/41.6
Higher education	69/54	63.3/53.5
Not stated	8/5	7.3/5.0
Employment^1^ (mothers/ fathers)		
Fully employed	11/94	10.1/93.1
Partly employed	54/4	49.5/4.0
No employment	42/3	38.5/3.0
Not stated	2/0	1.8/0
Social support^2^ (mothers/ fathers)		
Poor	23/13	21.1/12.9
Moderate	49/61	45.0/60.4
Strong	37/27	33.9/26.7

Patients	*n*	%
Patient's gender		
Female	44	38.6
Male	70	61.4
Patient receives level of care^3^		
Yes	43	37.7
No	71	62.3
Patient rare disease		
Anorectal malformation	30	26.3
Biliary atresia	14	12.3
Congenital diaphragmatic hernia	14	12.3
Esophageal Atresia	27	23.7
Hirschsprung’s disease	29	25.4

^1^Refers to the last 12 months. ^2^Social support is measured with the Oslo-Social Support Scale [23] and operationalized into three broad categories referring to [31]. ^3^Referes to the decision for the classification in the care insurance according to the German long-term care insurance

### Correlation analyses

Table 2 shows the Pearson correlation coefficients and level of possible associations between variables of the ABCX-Model separated for gender. Significant bivariate associations were found for mothers for all variables and stressor pile-up, except for family functioning. In contrast, significant bivariate associations were found for fathers all variables and stressor pile-up, except for social support. No significant associations were found for family functioning, social support, and perceived stress for both mothers and fathers.

**Table 2:** Pearson correlation between predictor and outcome parameters (*n* = 204)

Variables	1	2	3	4	5
Mothers (*n* = 107)					
1. Stressor Pile-Up [aA]	–				
2. Family Functioning (FAM) [bB]	0.169 (.080)	–			
3. Social Support (OSSS) [bB]	**−** **0.236 (.014)**	**−** **0.214 (.027)**	–		
4. Perceived Stress (IFS) [cC]	**0.427 (.001)**	0.119 (.220)	**−** 0.110 (.260)	–	
5. Mental Health (BSI GSI) [xX]	**0.297 (.002)**	**0.494 (.001)**	**−** **0.370 (.001)**	**0.298 (.002)**	–
*M*	2.90	24.56	10.30	2.11	0.49
*SD*	1.57	7.20	2.20	0.52	0.48
*Median*	3.00	23.50	10.00	2.00	0.28
*Min, max*	0, 7	14, 50	6, 15	1, 4	0, 3
Fathers (*n* = 97)					
1. Stressor Pile-Up [aA]	–				
2. Family Functioning (FAM) [bB]	**0.217 (.031)**	–			
3. Social Support (OSSS) [bB]	**−** 0.163 (.105)	**−** **0.377 (.001)**	–		
4. Perceived Stress (IFS) [cC]	**0.405 (.001)**	0.029 (.775)	**−** 0.172 (.088)	–	
5. Mental Health (BSI GSI) [xX]	**0.324 (.001)**	**0.385 (.001)**	**−** **0.278 (.006)**	**0.245 (.015)**	–
*M*	2.89	24.66	10.46	2.10	0.26
*SD*	1.61	7.54	1.70	0.37	0.26
*Median*	3.00	23.00	10.00	2.07	0.20
*Min, max*	0, 7	12, 48	5, 14	1, 3	0, 1

Bold values indicate statistical significance at the p < .05 level

*M*: Mean, *SD*: Standard deviation*, Min*: minimum score*, Max*: *maximum score.* Main entries are Pearson *r*, with *p* values in parenthesis. FAM: Family Assessment Measure, OSSS: Oslo Social Support Scale, IFS: Impact on Family Scale, BSI GSI: Brief Symptom Inventory Global Severity Index

### Mediation analyses

**Fig. 2:**
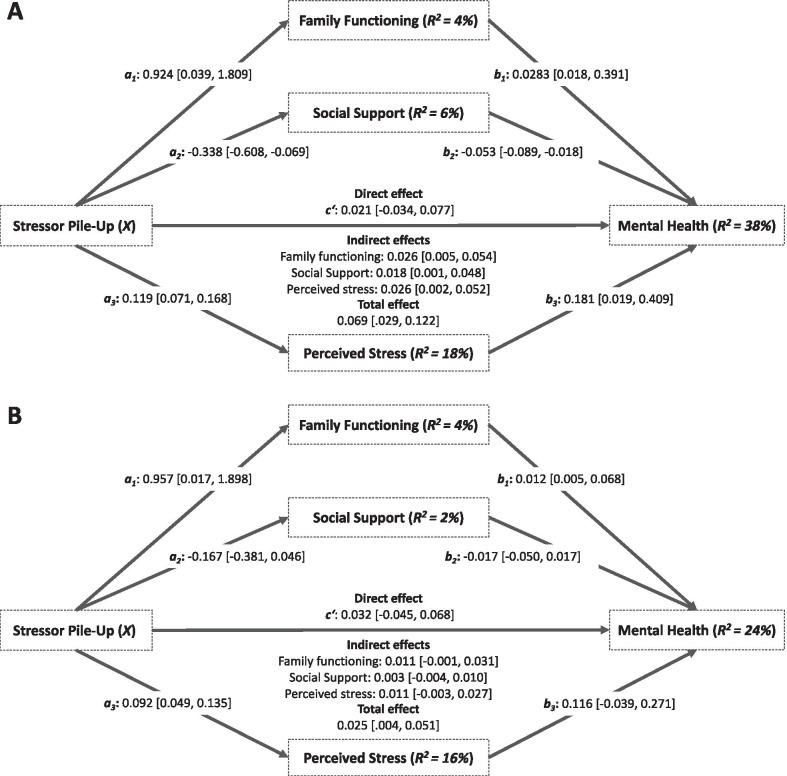
Parallel mediation of mothers (Model **A**) and fathers (Model **B**) of children with rare congenital surgical diseases. Note. The Path *a* represents the effect of stressor pile-up on each mediator; Path *b* represents the combined effects of each mediator on parental mental health; the direct effect represents the effect of the stressor pile-up on parental mental health while keeping levels of the mediators constant; the indirect effect represents the combined effect of path *a* and path *b* and therefore the mediation. The total effect represents the combined indirect and direct effects. Significance inferences at the α = .05 for effects are based upon the notion of whether confidence intervals include zero. FAM: Family Assessment Measure; OSSS: Oslo Social Support Scale; IFS: Impact on Family Scale; BSI GSI: Brief Symptom Inventory Global Severity Index

Table 3 and Fig. 2 show the multiple mediation models for mothers and fathers. Results showed significant indirect effects of stressor pile-up on mental health through family functioning, social support, and perceived stress in mothers (Model A; see Fig. 2). While the total effect of stressor pile-up on mental health was significant (*b* = 0.069, *p* = 0.003, *R*^2^ = 0.084), the direct effect was not statistically significant after including family functioning, social support, and perceived stress. The pattern of direct, indirect, and total effects suggests that family functioning, social support, and perceived stress fully mediated the association between stressor pile-up and mental health in mothers of children with rare congenital surgical diseases. The multiple mediator model nearly explained forty percent of the variance in perceived mental health in mothers.

**Table 3:** Multiple mediator models predicting parental mental health in mothers (*n* = 107) and fathers (*n* = 97)

Mediation Models	FAM (*M*_*1*_)					OSSS (*M*_*2*_)				IFS (*M*_*3*_)				BSI GSI (*Y*)			
		*b*	*SE*	*p*			*b*	*SE*	*p*		*b*	*SE*	*p*		*b*	*SE*	*p*
Mothers (*n* = 107)																	
Stressor pile-up (*X*)	*a*_*1*_	*0.924*	*0.447*	.041		*a*_*2*_	*− 0.338*	*0.136*	.014	*a*_*3*_	*0.119*	*0.025*	< .001	*c’*	*0.021*	*0.028*	.449
FAM (*M*_*1*_)		*-*	*-*	*-*			*-*	*-*	*-*		*-*	*-*	*-*	*b*_*1*_	*0.028*	*0.006*	< .001
OSSS (*M*_*2*_)		*-*	*-*	*-*			*-*	*-*	*-*		*-*	*-*	*-*	*b*_*2*_	*− 0.053*	*0.018*	.004
IFS (*M*_*3*_)		*-*	*-*	*-*			*-*	*-*	*-*		*-*	*-*	*-*	*b*_*3*_	*0.214*	*0.098*	.031
Constant	${i}_{{M}_{1}}$	*21.686*	*1.511*	< .001		${i}_{{M}_{2}}$	*11.321*	*0.460*	< .001	${i}_{{M}_{3}}$	*1.754*	*0.083*	< .001	${i}_{Y}$	*− 0.170*	*0.314*	.588
	*R*^*2*^ = .039					*R*^*2*^ = .056				*R*^*2*^ = .184				*R*^*2*^ = .379			
	*F*(1,105) = 4.283, *p* = .041					*F*(1,105) = 6.188, *p* = .014				*F*(1,105) = 23.685, *p* < .001				*F*(4,102) = 15.569, *p* < .001			
Fathers (*n* = 97)																	
Stressor pile-up (X)	*a*_*1*_	*0.957*	*0.474*	< .001		*a*_*2*_	*-0.167*	*0.107*	.124	*a*_*3*_	*0.092*	*0.022*	< .001	*c’*	*0.032*	*0.018*	.086
FAM (M_1_)		*-*	*-*	*-*			*-*	*-*	*-*		*-*	*-*	*-*	*b*_*1*_	*0.012*	*0.004*	.003
OSSS (M_2_)		*-*	*-*	*-*			*-*	*-*	*-*		*-*	*-*	*-*	*b*_*2*_	*− 0.017*	*0.017*	.328
IFS (M_3_)		*-*	*-*	*-*			*-*	*-*	*-*		*-*	*-*	*-*	*b*_*3*_	*0.116*	*0.078*	.140
Constant	${i}_{{M}_{1}}$	*21.727*	*1.614*	.046		${i}_{{M}_{2}}$	*10.813*	*0.367*	< .001	${i}_{{M}_{3}}$	*1.827*	*0.074*	< .001	${i}_{Y}$	*− 0.189*	*0.293*	.519
	*R*^*2*^ = .041					*R*^*2*^ = .025				*R*^*2*^ = .158				*R*^*2*^ = .237			
	*F*(1,95) = 4.081, *p* = .046					*F*(1,95) = 2.415, *p* = .124				*F*(1,95) = 17.865, *p* < .001				*F*(4,92) = 7.149, *p* < .001			

DV: dependent variable*, b*: unstandardized regression coefficient, *SE*: standard error, 95%-*CI*: 95% confidence interval. FAM: Family Assessment Measure, OSSS: Oslo Social Support Scale, IFS: Impact on Family Scale, BSI GSI: Brief Symptom Inventory Global Severity Index

Regarding fathers, results showed no significant indirect effects of stressor pile-up on mental health through family functioning, social support, and perceived stress in fathers (Model B; see Fig. 2). While the total effect of stressor pile-up on mental health was significant (*b* = 0.025, *p* = 0.002, *R*^2^ = 0.099), the direct effect was not statistically significant after including the respective mediator variables. The pattern of direct, indirect, and total effects suggests that family functioning, social support, and perceived stress all do not mediate the association between stressor pile-up and mental health in fathers of children with rare congenital surgical diseases. Nonetheless, nearly one-fourth of the variance in perceived mental health in fathers was explained by the multiple mediator model.

## Discussion

The present study used the double ABCX model as a theoretical framework to investigate the associations between stressor pile-up, resources, and mental health in mothers and fathers of children with rare congenital surgical diseases. For affected mothers, our results were consistent with previous research on chronic childhood conditions, demonstrating a significant association between parental stressor pile-up and adaptation, and a decrease of this association by appropriate internal and external resources, and perceived ability to effectively deal with stress [15–20].

Most importantly, our findings contribute to understanding the association between parental stressors predominantly due to the child’s disease and the current mental health, emphasizing the mediating role of family-related resources and perceived stress. Our multiple mediation supports this conclusion in mothers, but not fathers. Family functioning, social support, and perceived stress were all found to fully mediate the association between parental stressor pile-up and mental health in mothers, whereas, in fathers, the results indicate a direct-only non-mediation [32]. These findings support the proposition that when mothers experience increased exposure to stressors, they may respond adequately to these crisis events through available internal and external resources as well as the appraisal of the situation [33]. Therefore, the approach of using the ABCX model in the context of parents of children with rare congenital surgical disease seems to be confirmed for affected mothers but not for fathers.

It is also important to note that it was found that mothers of children with rare congenital surgical diseases reported higher levels of overall psychological distress compared to fathers, which is in line with previous research on parents of children with chronic conditions [10, 34] and the general adult populations [35, 36]. In addition, mothers, compared to fathers, are typically more involved in taking care of their children. Therefore, it may be that mothers feel more responsible for the illness, and consequently, the caregiving burden increases, which could explain additional increased parental psychosocial impairment in mothers compared to fathers.

In this study, responding parents had a higher education primarily and were married; thus, parents in these families may have had an additional protective factor of financial resources and having a stable primary source of social support in contrast to the general population [33].

In particular, our results confirm the importance of intervening factors in explaining parental stress in fathers and mothers caring for a child with rare congenital surgical diseases. Although the framework within the ABCX model could not be confirmed for fathers, preliminary correlation analysis showed that there were associations among the included variables. Additional factors would have to be investigated in future studies to determine more relevant variables for paternal mental health within the ABCX model. The identification of potential protective and risk factor such as marital satisfaction and support from the partner may be more suitable variables in the theoretical framework, as previous qualitative research suggests, and related to this, father-friendly programs may be enhanced through the focus on the parent’s partnerships [37].

## Study limitations

In the present study, several limitations have to be recognized. First, the representativeness of the sample may be somewhat limited regarding the age of the affected children. Since participating families had considerably younger children than non-participating families, the possibility of non-response bias cannot be ruled out. Thus, the psychosocial impairment of affected parents may be overestimated, as it could be argued that parents of older children are less impaired. Nevertheless, the participation rate was similar to previous research on child health in western countries [38, 39]. Second, our cross-sectional multiple mediation analyses do not allow for causal interpretations, even if the pile-up of the different components of the stressor may be justified as a causal factor. Third, any conclusion cannot be drawn on clinical groups other than the rare congenital surgical diseases presented in this study. Fourth, the psychometric instruments used were not validated on populations such as rare diseases, so this must be considered when interpreting the results. Lastly, although rare congenital surgical conditions are present within the studied sample and parents may adapt better over time, these rare conditions may increase in childhood due to comorbidities and complications and lead to a renewed increase in psychological distress for parents. Unfortunately, information on these factors was not collected in the questionnaires because of the cross-sectional study design. Therefore, further detailed research is needed using longitudinal designs in which comprehensive disease-specific and psychosocial outcomes are collected prospectively from large, multi-center samples using rigorous analyses. This is the case in a recent multi-center study examining two psychosocial interventions within Germany [40].

## Conclusion

To our knowledge, the Double ABCX model has not been used to investigate the mental health of parents caring for children with rare congenital rare diseases, even if this has already been investigated in the context of children's chronic diseases [15–18, 20]. Our findings contribute to the literature on parental adaptation to their children's rare congenital surgical disease by describing the gender-specific functioning of the affected parents. Potential areas for family intervention can thus be identified to promote resilience in affected parents. Future research should incorporate prospective, longitudinal studies to further describe the qualities that contribute to parental adjustment to rare congenital surgical disease so that within specific family-oriented interventions, these qualities can be promoted.

## Data Availability

The datasets generated during the current study are available from the corresponding author on reasonable request.

## Abbreviations

BSI: Brief symptom inventory
FAM: Family assessment measure
GSI: Global severity index
OSSS: Oslo social support scale
SDQ: Strength and difficulties questionnaire
IFS: Impact on family scale
